# High-probability heart failure with preserved ejection fraction phenotype in atrial fibrillation patients undergoing ablation: insights from RECAP

**DOI:** 10.1093/europace/euag211

**Published:** 2026-08-11

**Authors:** Jonas Erzeel, Michiel De Wever, Marnicq van Es, Sebastiaan Dhont, Wilfried Mullens, Laurent Pison, Pieter Martens

**Affiliations:** Department of Cardiology, Ziekenhuis Oost-Limburg, Synaps Park 1, 3600 Genk, Belgium; Faculty of Medicine and Life Sciences/LCRC, Hasselt University, Building D, Agoralaan, 3590 Diepenbeek, Belgium; Department of Cardiology, Ziekenhuis Oost-Limburg, Synaps Park 1, 3600 Genk, Belgium; Faculty of Medicine and Life Sciences/LCRC, Hasselt University, Building D, Agoralaan, 3590 Diepenbeek, Belgium; Department of Cardiology, Ziekenhuis Oost-Limburg, Synaps Park 1, 3600 Genk, Belgium; Faculty of Medicine and Life Sciences/LCRC, Hasselt University, Building D, Agoralaan, 3590 Diepenbeek, Belgium; Department of Cardiology, Ziekenhuis Oost-Limburg, Synaps Park 1, 3600 Genk, Belgium; Faculty of Medicine and Life Sciences/LCRC, Hasselt University, Building D, Agoralaan, 3590 Diepenbeek, Belgium; Department of Cardiology, Ziekenhuis Oost-Limburg, Synaps Park 1, 3600 Genk, Belgium; Faculty of Medicine and Life Sciences/LCRC, Hasselt University, Building D, Agoralaan, 3590 Diepenbeek, Belgium; Department of Cardiology, Ziekenhuis Oost-Limburg, Synaps Park 1, 3600 Genk, Belgium; Faculty of Medicine and Life Sciences/LCRC, Hasselt University, Building D, Agoralaan, 3590 Diepenbeek, Belgium; Department of Cardiology, Ziekenhuis Oost-Limburg, Synaps Park 1, 3600 Genk, Belgium; Faculty of Medicine and Life Sciences/LCRC, Hasselt University, Building D, Agoralaan, 3590 Diepenbeek, Belgium

**Keywords:** Atrial Fibrillation, Heart failure with preserved ejection fraction, Catheter ablation, HFpEF-ABA score, Underdiagnosis

## Introduction

Heart failure with preserved ejection fraction (HFpEF) frequently coexists with atrial fibrillation (AF), sharing metabolic, structural, and haemodynamic substrates.^1^ However, HFpEF remains systematically underdiagnosed in AF populations, with invasive haemodynamic studies suggesting a higher prevalence than clinically recognized.^2^ Diagnosis is challenging due to symptom overlap, unreliable echocardiographic assessment of diastolic dysfunction in AF, and elevation of natriuretic peptides in AF independent of HFpEF.^1^ Patients referred for AF ablation represent a population enriched for obesity, hypertension, and atrial cardiomyopathy, in whom unrecognized HFpEF may be particularly prevalent and clinically relevant.^3^ We therefore evaluated the prevalence of a HFpEF phenotype using the HFpEF-ABA score in a retrospective single-centre AF ablation cohort, and examined associated features, treatment patterns, and exploratory ablation outcomes.

## Methods

Data was analysed from the Retrospective Comparison of Ablation Procedures for Early Recurrent Atrial Tachyarrhythmias (RECAP) study, including consecutive patients undergoing catheter ablation for symptom-refractory AF between September 2024 and June 2025. Following ablation, patients initiated rhythm monitoring using the Fibricheck smartphone application. Patients with left-ventricular ejection fraction ≥50% and available data required for HFpEF-ABA score calculation were included. The study was approved by the local ethics committee, with informed consent waived due to the retrospective design.

The HFpEF-ABA score is a validated logistic regression-based model incorporating age, body mass index (BMI), and history of AF to estimate HFpEF probability.^4^ Individual probabilities were calculated, with ≥90% classified as high-probability HFpEF. Patients were subsequently categorized into three mutually exclusive groups: low-probability HFpEF, clinically diagnosed HFpEF (defined as a prior physician-documented diagnosis in the medical record, reflecting routine clinical assessment), and high-probability HFpEF without a documented HFpEF diagnosis.

Fibricheck is a Ce-marked, smartphone-based photoplethysmography application enabling intermittent rhythm assessment and automated AF detection.^5^ Patient were instructed to perform 60-s measurements twice-daily and additional symptom-triggered recordings. AF recurrence was defined as any documented AF episode occurring after an 8-week blanking period and within 6 months of follow-up. Time-to-first AF recurrence was used for survival analyses.

Continuous variables are presented as mean ± standard deviation and compared using the Kruskal–Wallis test. Categorical variables are presented as counts (percentages) and compared using the *χ*² test. Atrial fibrillation recurrence was analysed using Kaplan–Meier curves and Cox proportional hazards regression. Analyses were exploratory, with *P* < 0.05 considered statistically significant.

## Results

**Figure 1:**
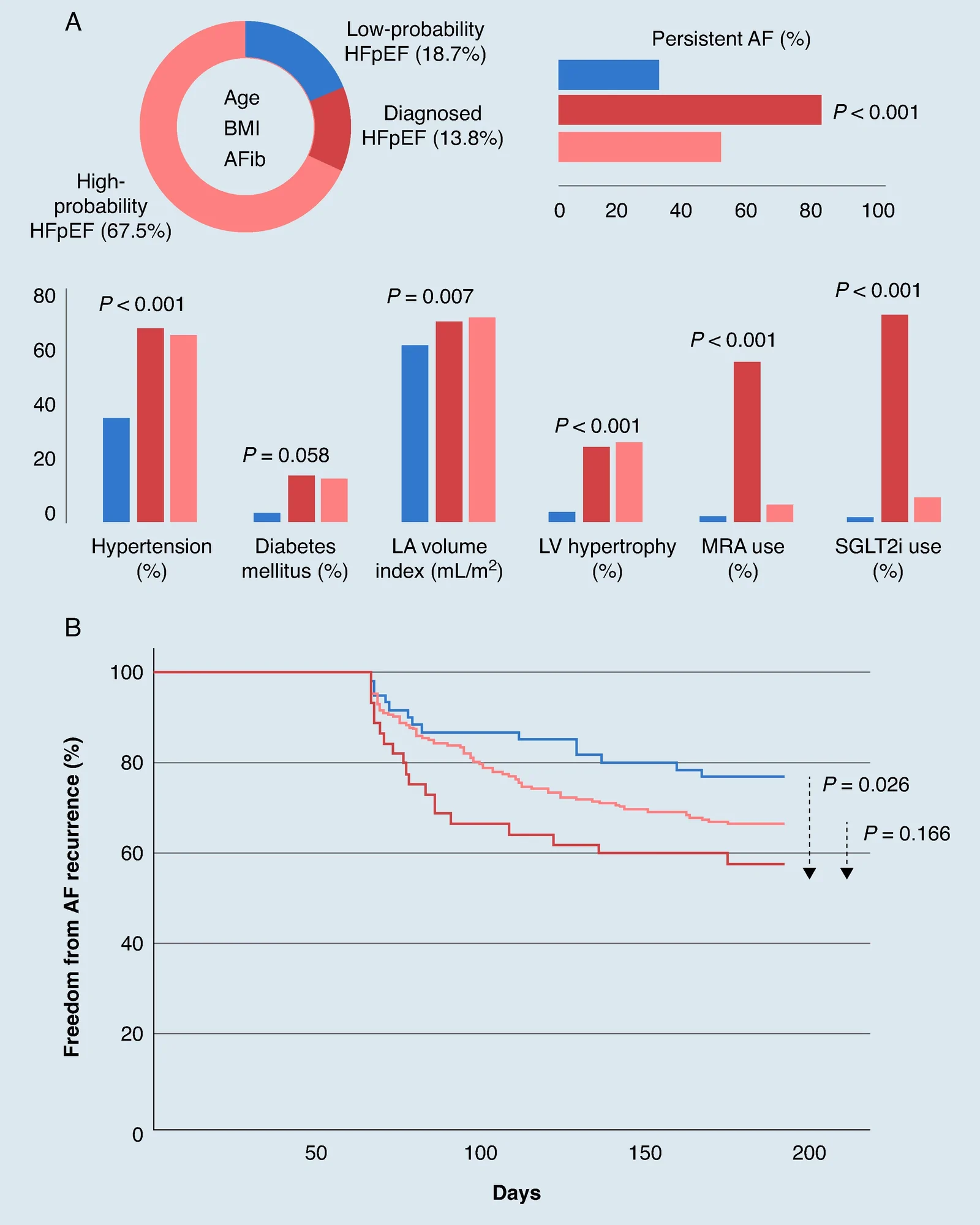
Clinical profile and AF recurrence across HFpEF groups. (*A*) Distribution of HFpEF groups and selected comorbidities, structural parameters, and HFpEF-directed therapies. (*B*) Kaplan–Meier curves for freedom from AF recurrence by HFpEF group.

Among 326 patients undergoing AF ablation, 45 (13.8%) had a documented HFpEF diagnosis. In contrast, the HFpEF-ABA score identified 259 patients (79.4%) with a high probability (≥90%) of HFpEF. Patients were classified as low-probability HFpEF (*n* = 61 [18.7%]), clinically diagnosed HFpEF (*n* = 45 [13.8%]), or high-probability HFpEF without a documented diagnosis (*n* = 220 [67.5%]; *Figure 1A*).

Patients with clinically diagnosed and high-probability HFpEF were older and had higher BMI than those with low-probability HFpEF. Beyond these score-derived differences, both groups demonstrated a distinct cardiometabolic phenotype with higher prevalence of hypertension (64.4% and 62.7% vs. 32.8%; *P* < 0.001), diabetes mellitus (15.6% and 14.1% vs. 3.3%; *P* = 0.058), and obstructive sleep apnoea (OSAS) (11.1% and 18.2% vs. 6.6%; *P* = 0.058) compared to those with low-probability HFpEF.

Persistent AF was more common in clinically diagnosed (82.2%) and high-probability HFpEF (50.5%) than in low-probability HFpEF (29.5%; *P* < 0.001). Left-atrial volume index (by computed tomography) was higher (67.0 ± 19.3 and 67.7 ± 19.4 vs. 58.1 ± 21.3 mL/m^2^; *P* = 0.007), and left-ventricular hypertrophy (LVH) more prevalent (25.0% and 26.7% vs. 3.4%; *P* < 0.001) in both HFpEF groups.

Treatment differed substantially. Mineralocorticoid receptor antagonists (53.3% vs. 4.1% and 1.6%; *P* < 0.001), sodium–glucose cotransporter-2 inhibitors (68.9% vs. 5.5% and 1.6%; *P* < 0.001), and loop diuretics (13.3% vs. 3.2% and 0.0%; *P* = 0.001) were prescribed more frequently in clinically diagnosed HFpEF than in high-probability and low-probability HFpEF groups.

Cox regression showed a higher AF recurrence risk in clinically diagnosed HFpEF compared with low-probability HFpEF (HR 2.20; 95% CI 1.10–4.38; *P* = 0.026), while no difference was observed between clinically diagnosed and high-probability HFpEF (*P* = 0.166). Kaplan–Meier curves demonstrated a graded pattern across groups (*Figure 1B*).

## Discussion

In this retrospective AF ablation cohort, the HFpEF-ABA score identified a large group of patients with a high-probability HFpEF phenotype without clinical diagnosis. Importantly, these patients demonstrated a clinical and structural profile similar to diagnosed HFpEF, including higher prevalence of hypertension, diabetes, OSAS, atrial remodelling, and LVH, suggesting that HFpEF may be substantially under-recognized in AF populations.

Marked differences in HFpEF-directed therapy further underscore this gap. Despite comparable phenotype severity, patients with high-probability HFpEF without a documented diagnosis rarely received disease-modifying therapies. These findings suggest potential opportunities for HFpEF-directed therapies and comorbidity management, although implications for residual symptoms, functional limitations, and AF recurrence remain hypothesis-generating. More systematic screening for HFpEF and cardiometabolic comorbidities prior to AF ablation may help identify high-risk patients and optimize management.

Exploratory analyses showed a graded pattern of AF recurrence, highest in clinically diagnosed HFpEF, intermediate in high-probability HFpEF, and lowest in patients with low-probability HFpEF. Cox regression confirmed higher recurrence risk in clinically diagnosed HFpEF vs. low-probability HFpEF, with no separation from high-probability HFpEF. These findings align with prior observations linking HFpEF to increased AF recurrence after ablation, likely reflecting progressive atrial cardiomyopathy and substrate-driven AF.^6^ The higher prevalence of persistent AF in clinically diagnosed HFpEF may have contributed to recurrence risk, although this may also reflect underlying HFpEF-related atrial remodelling.

### Study limitations

This study is observational and exploratory, with a limited sample size and unequal group distributions, which restrict statistical power for detecting group differences, particularly for outcome analyses such as AF recurrence. Heart failure with preserved ejection fraction probability was derived from a clinical score rather than invasive haemodynamic assessment, echocardiography or biomarker confirmation. Furthermore, prior studies have demonstrated discordance between HFpEF risk scores and invasive haemodynamics, underscoring both the limitations of score-based classification and the complexity of HFpEF diagnosis in AF populations.^7^ As a result, outcome findings should be interpreted as hypothesis-generating.

## Conclusions

In patients undergoing AF ablation, potentially unrecognized HFpEF is common and mirrors clinically diagnosed HFpEF in structural and metabolic features, yet remains undertreated, highlighting an opportunity to improve HFpEF recognition and management.

## Data Availability

The data underlying this article cannot be shared publicly due to the privacy of individuals who participated in the study. De-identified data will be shared on reasonable request to the corresponding author.
